# Supplementary material for: The interactome of intact mitochondria by cross-linking mass spectrometry provides evidence for coexisting respiratory supercomplexes
Source: Mol Cell Proteomics. 2017 Dec 8;17(2):216–32. doi: 10.1074/mcp.RA117.000470 (PMC5795388; doi:10.1074/mcp.RA117.000470)
Supplement: Supplemental Data [file supp_RA117.000470_133922_0_supp_23978_fzffwf.zip › spectra_annotation/mito_spectra_annotation/433-1-6-1-4-1.pdf]

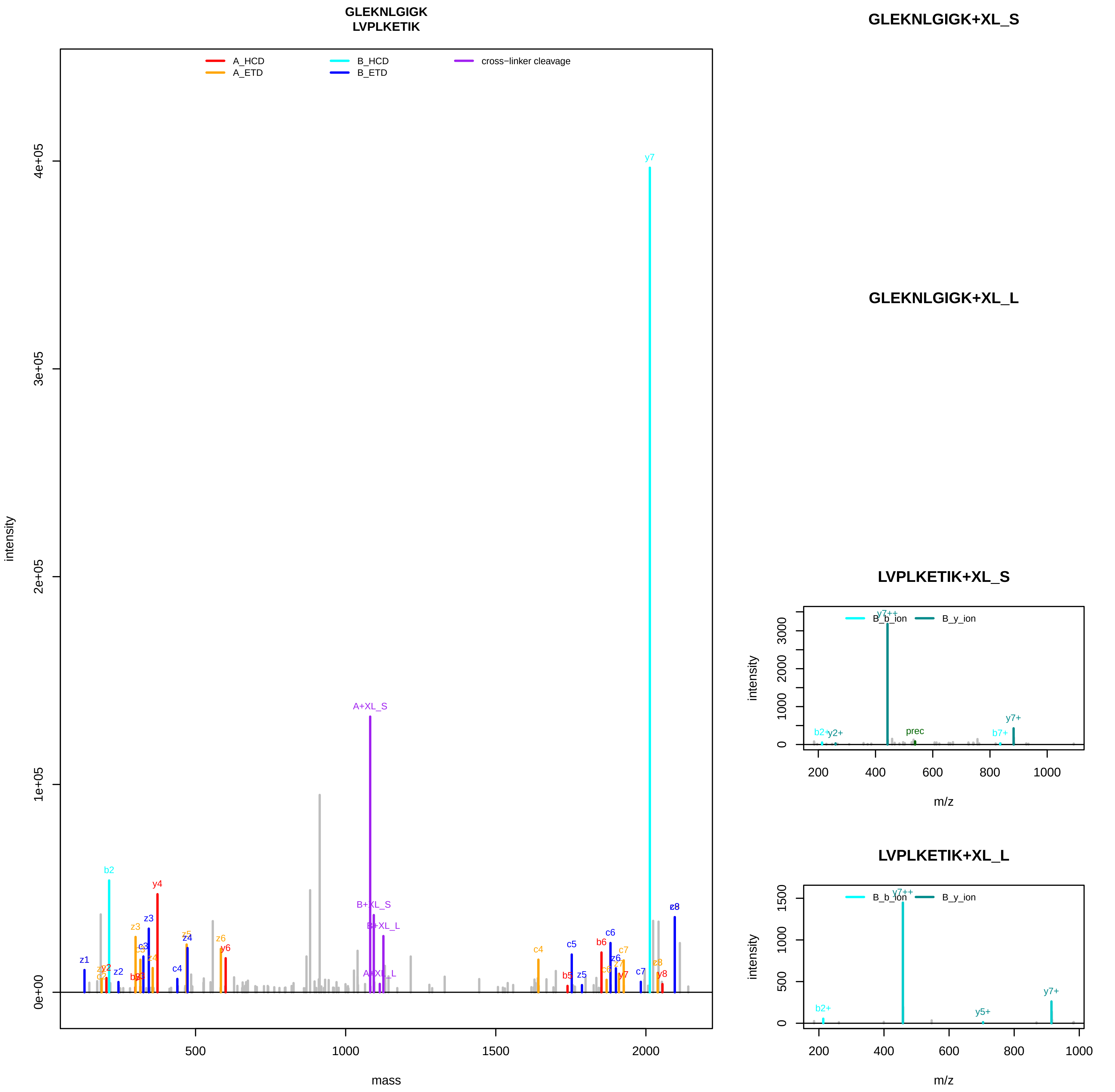

GLEKNLGIGK
LVPLKETIK
A_HCD
A_ETD
B_HCD
B_ETD
cross−linker cleavage
intensity
mass
y7
A+XL_S
B+XL_S
B+XL_L
A+XL_L
b2
y4
z1
z2
z3
c3
z4
c4
z5
z6
y6
c4
c5
b5
b6
c6
c7
y7
z8
y8
GLEKNLGIGK+XL_S
GLEKNLGIGK+XL_L
LVPLKETIK+XL_S
B_b_ion
B_y_ion
y7++
b2+
y2+
prec
b7+
y7+
m/z
LVPLKETIK+XL_L
y5+
